# Relationship between the abdominal sagging index and the reproductive performance of the Roman goose

**DOI:** 10.5713/ab.22.0230

**Published:** 2022-09-07

**Authors:** S. C. Chang, M. J. Lin, L. J. Lin, S. Y. Peng, Tzu Tai Lee

**Affiliations:** 1 Kaohsiung Animal Propagation Station, Livestock Research Institute, Council of Agriculture, Executive Yuan, Pingtung 91201, Taiwan; 2 Changhua Animal Propagation Station, Livestock Research Institute, Council of Agriculture, Executive Yuan, Changhua 52149, Taiwan; 3 School of Chinese Medicine, College of Chinese Medicine, China Medical University, Taichung 40402, Taiwan; 4 Department of Animal Science, National Pingtung University of Science and Technology, Pingtung 91201, Taiwan; 5 Department of Animal Science, National Chung Hsing University, Taichung 40227, Taiwan; 6 The iEGG and Animal Biotechnology Center, National Chung Hsing University, Taichung, 40227, Taiwan; 7 Smart Sustainable New Agriculture Research Center (SMARTer), Taichung 40227, Taiwan

**Keywords:** Abdominal Sagging Index, Reproductive Performance, White Roman Goose

## Abstract

**Objective:**

This research aimed to explore the changes in the observed abdominal sagging index (ASI) and reproductive performance of Roman male and female geese during the breeding period.

**Methods:**

The 339 six-month-old breeding geese (109 male; 230 female) were used in this study, in which five male and five female geese were slaughtered on a monthly basis to record the ASI.

**Results:**

The short diameter of the testes of the male goose when the female goose lays eggs and in the second, third, and fourth months was significantly wider than in the fifth months (19.0, 20.8, 21.4, and 19.6 vs 12.7 and 14.0 mm/bird; p = 0.0105). On the other hand, the testicular weight of the male goose in the second and third months after the female goose lays eggs was significantly higher than that in the second and fifth months after laying (0.33% and 0.37% vs 0.11% and 0.19%; p = 0.0212). During the exploring period, the length and weight of the fallopian tube, the weight of the ovary, the number of follicles in 2 to 3 cm, the number of follicles in 3 to 4 cm, the fallopian tube weight in the carcass weight percentage, and the ovary weight in the carcass weight percentage all demonstrated a significant curve response. Further, female ASI was positively correlated with reproductive tract length (r = 0.815; p<0.05) and egg production per female (r = 0.790; p<0.05).

**Conclusion:**

The ASI classification method is more objective and easy to distinguish. This scoring method has a high correlation with the number of eggs laid by each goose and the length of the reproductive tract, inferring that the goose observation could take advantage of ASI during egg-laying and can predict the reproductive system development during the laying period and determine when the breeding goose begins to lay eggs.

## INTRODUCTION

Geese are the oldest domesticated poultry species whose domestication dates back 7,000 years [1] and they are seasonal breeding animals regulated by natural light [2]. Taiwan is located in a subtropical region with high temperatures and humidity. In the Changhua county, for instance, the egg-laying period of female geese in the natural environment is approximately from the beginning of October to the end of May of the following year, and the sunshine time (between sunrise and sunset) is 11 hours and 56 minutes and 13 hours and 31 minutes, respectively. However, during the egg-laying period of female geese, the sunshine time is at its shortest, at 10 hours and 39 minutes on December 22 [3]. Under natural conditions, the egg-laying rate of female geese shows a curve reaction. Influenced by sunlight and temperature in the natural environment, the egg-laying period of Taiwanese female geese is from the beginning of October to the end of May of the following year. In addition, the productive period is from January to March, while the rest period is from June to September [4].

The proportion of goose weight loss may influence the uniformity of laying geese in the next laying period [4]. Bogenfürst et al [5] suggested that feeding geese should be restricted to reduce their body weight to less than 4.75 kg, which can increase the number of eggs laid per goose and the fertilization rate of eggs in the next laying period. Therefore, breeding geese are given restricted rations during and before the laying period, mainly to avoid over-fat geese and delay their production. Breeding geese weigh approximately 3.5 kg during the off-production period and can reach 4.9 kg when fed with egg-laying material from the start of production to the peak of egg-laying. In a study, the body weight of primiparous female geese was 3.27 to 3.83 kg during the rest period, 4.78 to 5.01 kg at the peak egg production, and 4.61 to 5.11 kg at the end of the experiment [6]. The above data demonstrated that breeding geese should be fed at an appropriate time during the laying off period to control their weight, considering that excess and lack of weight may influence their egg-laying performance during the laying period.

In high-yielding geese the serum estradiol, glucose, and triglyceride concentrations are high and they have significant pubic spacing and abdominal circumference [7]. The body scoring method is applied to waterfowl and swans, which allows one to avoid grasping animals to repeatedly check the weight of individual animals and other data. In addition, it roughly examines the animal’s current weight and internal tissue changes to predict when the breeding geese start to lay eggs [8,9].

The body weight, muscle mass, and tibial parameters of geese after 28 d vary according to sex [10]. Lipid and protein stores are determined as an index of body condition in arctic geese. Protein stores are more stable than lipids, which change with migration. The geese could increase endogenous lipid stores when they intake enough food resources [11]. Further, Pacific greater fronted geese have a nonlinear change in body conditions. They adapt to their migration area and adjust their body conditions accordingly [12]. A study found that gandersin the breeding stages had heavier testicular weights than those of the non-breeding stages [13]. According to Madsen and Klaassen’s abdominal profile index (API) scoring method, it is possible to distinguish the body changes of breeding geese during the laying process. Notably, the API range of pink-footed geese can range from 0 to 7 points. Further, animal physiology and nutritional status are related to body and organ weights. Landers et al [14] suggested that the heart and liver weights of geese treated with restricted feeding are significantly lighter than those in the free-feeding group. With adequate nutrition, geese gradually gain weight and accumulate body fat for reproduction and growth. Hence, body part percentages and organ weights change accordingly. Before the breeding season, geese need to gradually accumulate their energy prior to breeding. During the breeding season, its reproductive system develops with the season and physiology. Once laying commences the weight of the chicken and its oviduct and ovary are heavier [15,16]. On the other hand, tissue development in turkeys prior to laying has demonstrated that oviducts develop faster than ovaries by 3 days [17]. On the male side, the penis size of various poultry species varies greatly—the ostrich’s penis length is 30.1 cm, and its weight is 870 g [6]; the male duck’s penis length is 22.3 cm, and its penis weight is 10.8 g [18]. Some waterfowl species are seasonal animals, and there is little information on the development status before and after the breeding season. In this study, the goose body posture method was utilized to evaluate the reproductive system development during egg-laying to uncover the correlation between the observation index and the reproductive system and predict the reproductive system changes of the goose during the egg-laying period.

## MATERIALS AND METHODS

### Goose flock and sampling

The experimental protocol was approved by the Animal Care and Use Committee of Changhua Animal Propagation Station, Livestock Research Institute, Executive Yuan, Changhua, Taiwan (IACAC-CH9702). This research employed commercial White Roman goslings born on April 15 and cooped on October 20 of the same year. At the beginning of the survey, 339 6-month-old breeding geese (109 male; 230 female) were used, in which five male and five female geese were slaughtered every month from the beginning of the breeding season in October to the end of the breeding season in May of the following year. In particular, the five male and five female geese were utilized to detect the weight of their carcasses and reproductive systems. A total of 40 male and 40 female geese are slaughtered. Further, the abdominal sagging index (ASI) of other male and female geese were detected, and the egg-laying data of geese were also recorded. This batch of breeding geese would start to lay eggs on December 10, 1999. Therefore, December is the starting month of egg-laying, while November of the same year is the first month before egg-laying and the following month after egg-laying (January of the following year), and it is the first month of the laying period, and so on.

### Feeding management

The geese in this study were fed individually. The female goose coop was 40 cm wide (W), 50 cm deep (L), and 65 cm high (H). On the other hand, the male goose coop was 50 cm wide (W), 70 cm deep (L), and 80 cm high (H). During the resting period, the geese were given 150 g of resting ration per day, with a crude protein content of 13% and a metabolizable energy content of 2,350 kcal/kg. During the laying period, the geese were given a laying ration containing 15% or 18% crude protein and metabolizable energy of 2,350 kcal/kg. At this stage, the geese were only given a laying ration.

The sanitation and epidemic prevention plan was implemented in accordance with the specifications set by the Changhua animal propagation farm, and the goose cages and utensils were regularly disinfected. The breeding geese were given an intramuscular injection of poultry cholera vaccine once at 4 and 8 weeks of age, respectively. In addition, two intramuscular injections of waterfowl parvovirus vaccine were completed one month before egg-laying, and the interval between the two injections was four weeks. Two months after the end of the laying period, the breeding geese were given an intramuscular injection of poultry cholera vaccine.

### Reproductive gland analysis

During the study period, five female geese were slaughtered every month, and their carcass traits, including pre-slaughter body weight, reproductive tract length, ovary weight, and a number of follicles, were measured. The number of follicles was divided into five types: their diameters were 1 to 2 cm, 2 to 3 cm, 3 to 4 cm, 4 to 5 cm, and larger than 5 cm. The ovaries were taken out at the time of slaughter, and size and number of ovarian follicles were measured. The length and weight of the reproductive tract referred to that from the fimbria to the cloaca opening. On the other hand, ovarian weight referred to the weight of ovarian follicles. Five male geese were slaughtered every month, and their carcass traits, including pre-slaughter weight, testicular weight, testicular length, and penis length and weight, were measured.

### Establish an abdominal sagging index

The monthly API measures the body shape of the slaughtered geese before slaughtering. The methods used were API and ASI. API is an 8-point scale established by Madsen and Klaassen [8]. It is scored according to the degree of abdominal depression and protrusion of pink-footed geese. Meanwhile, the ASI is a 6-point system, scored by observing the abdomen from the side of the goose where the abdomen falls on the feet, which is more objective. The goose’s abdomen sags to above the knee joint (1 point), the abdomen sags to the knee joint (2 points), the abdomen sags to the knee joint and the knee joint to 1/2 of the toe joint (3 points), the abdomen sags to the knee joint and the toe joint 1/2 point (4 points), the abdomen drooping to the knee joint and the toe joint 1/2 to the toe joint (5 points), and the abdomen drooping to the toe joint (6 points).

### Statistical analysis

The data obtained from the survey were statistically analyzed with the Statistical Analysis System [19], and the variance analysis was performed with its general linear model procedure. Then, the least-square means (LSMEANS) method was used to determine the difference between the means of each treatment. The difference is significant. In this study, the data of male and female geese were analyzed separately, and the main effect was treated as the main effect for eight months before and after laying eggs. The mathematical model of the statistical analysis is as follows:


$${\text{Y}}_{\text{ijk}}=\mu +{\text{M}}_{\text{i}}+\gamma_{\text{ij}}$$

In the formula, Y_ijk_ represents the observed value of the jth geese processed in the ith month; μ represents the mean of all observations; M_i_ indicates the fixed effect of the ith month treatment (i = 1, 2, 3, 4, 5, 6, 7, 8); γ_ij_ represents the chance difference with a goose as the survey unit, and γ_ij_∩N (0, σ^2^γ).

The measured male goose weight, testicular weight and length, penis weight and length, and the female goose reproductive tract weight and length, ovary and follicle weight, and the number of follicles and other traits are analyzed by MANOVA.

## RESULTS

### Reproduction system of breeding geese

The outcomes of this research demonstrated that there was no significant difference in the body weight of the male goose before and after the laying period (Table 1). This research also illustrated that the testicular weight of male geese in the third month after commencement of the egg laying season tended to be heavier than those in the second and first months before and the fifth month after female geese laid eggs (18.5 vs 5.70, 10.6, and 8.32 g/bird; p = 0.0504) (Table 1) in a curvilinear relationship (p<0.01). The short diameter of the testes of the male goose when the female goose started laying eggs and in the second, third, and fourth months after the female goose laid eggs was significantly wider than that of the female goose in the second month before and the fifth month after the female goose laid eggs (19.0, 20.8, 21.4, and 19.6 vs 12.7 and 14.0 mm/bird; p = 0.0105). On the other hand, the testicular weight of the male geese in the second and third months after laying eggs was significantly higher than that of female geese in the second and third months after laying eggs. The second and the fifth months after the goose laid eggs was higher (0.33% and 0.37% vs 0.11% and 0.19%; p = 0.0212), illustrating a curvilinear relationship (p<0.01).

Furthermore, the results of this research demonstrated that the length of the oviduct of the female goose at the time of laying and the first, second, and third months after laying was significantly longer than that in the second month before laying and the fourth month after laying (62.0, 83.7, 78.7 and 69.8 vs 20.1 vs 30.4 cm/bird; p<0.001) (Table 1), implying a quadratic equation (p<0.001). The oviduct weight of female geese in the first and second months after laying was significantly higher than that in the second and first months before and third and fourth months after laying (107 and 181 vs 4.16, 27.6, 9.76, and 23.1 g/bird; p<0.001), implying a quadratic equation (p<0.001). The ovary weight of female geese in the first and second months after laying was significantly higher than that in the second month before and the third, fourth, and fifths month after laying (128 and 158 vs 3.24, 9.38, 4.50, and 29.5 g/bird; p = 0.0015), implying a quadratic equation (p<0.01). The percentage of oviducts in the carcass weight of female geese in the second month after laying was significantly higher than that in other months (p = 0.0039), implying a quadratic equation (p<0.05).

### Gander and goose on abdominal sagging index

The API of female geese in the first and second months after laying was significantly higher than that in the first and second months before laying and at the time of laying (5.89, 5.90 vs 5.36, 5.64, and 5.66) (Table 2). The ASI was significantly higher at laying, first, second, and third months after laying than at the first and second months before laying and during laying (3.89, 3.86, and 3.94 vs 3.34, 3.39, and 3.54).

### Relation of the abdominal sagging index and the reproduction system in gander

The male goose API in this research is positively correlated with ASI (r = 0.93; p<0.0001) (Table 3), and its testicular weight is positively correlated with the short testicular diameter (r = 0.90; p<0.001). In addition, there is a positive correlation trend between the long axis of testicular balls and the short diameter of testicular balls (r = 0.63; p<0.1). Further, the short diameter of goose testis is positively correlated with penis length (r = 0.68; p<0.1).

### Relation of the abdominal sagging index and the reproduction system in goose

The body weight and reproductive tract length (r = 0.805; p<0.05), ovarian weight (r = 0.877; p<0.01), 2 to 3 cm follicle number (r = 0.878; p<0.01), 3 to 4 cm and follicle number (r = 0.848, p<0.01) and 4 to 5 cm follicle number (r = 0.837; p<0.01) are positively correlated (Table 4). Further, API and ASI (r = 0.840; p<0.01) and the reproductive tract length (r = 0.786; p<0.05) are also positively correlated. Female ASI is positively correlated with the reproductive tract length (r = 0.815; p<0.05) and the egg production per female (r = 0.790; p<0.05). The reproductive tract length and reproductive tract weight of female goose (r = 0.716; p<0.05), ovarian weight (r = 0.732; p<0.05), number of follicles 2 to 3 cm (r = 0.905; p<0.01) and 3 to 4. Further, there is a positive correlation between the number of cm follicles (r = 0.856; p<0.01) and between the reproductive tract weight and ovarian weight (r = 0.930; p<0.001). There is also a positive correlation between the goose ovary weight and the follicle number of 2 to 3 cm (r = 0.696; p<0.05) and 3 to 4 cm (r = 0.711; p<0.05).

## DISCUSSION

Hocking and Duff [20] suggested that the excess body weight of males could influence the fertilization rate of their eggs. The results of this study differ from the above studies that infer that the body weight of breeding geese should be controlled during the laying off period. Hence, the feeding method of the male goose during the laying off period needs to be adjusted, and the weight of the breeding male goose should be controlled below 4.75 kg. Although the weight control of the breeding male goose during the laying off period can influence the reproductive performance of the laying goose, this aspect still requires further research. The weight of the male geese in this survey reached 5.25 kg before the breeding season, demonstrating that the feeding restriction rhythm during the lay-off period has not reached the expected target.

The increase in light stimulation during the growing period of broiler males could significantly increase the testicular weight, follicle-stimulating hormone, and testosterone content. However, there is no regression relationship between testicular weight and body weight, mainly because the testicular weight of cocks has a significant variation (i.e., 4 to 5 kg–rooster’s testicles weigh 0.9 to 44 g) [21]. The long and short diameters of the left testis of Argentine lake duck are 37 to 45 mm and 15 to 20 mm, respectively, and the long and short diameters of the right testis are 37 to 43 mm and 14 to 22 mm, respectively. The testicular weight is 10.8 g, accounting for about 1.7% of the body weight. McCracken [18] suggested that the penis length of a male duck is 22.3 cm. However, the current literature has not yet compared the changes in the developmental process of the reproductive system throughout the breeding season. Gumułka et al [13] indicated that the right testis weight at the onset of laying weighs heavier than in the non-breeding stage (6.8 g vs 1.7 g, p<0.05). The left testis weight at the onset of laying and breeding (April) weighed heavier than in the non-breeding stage (9.8 g and 8.2 g vs 1.7 g). The serum sex hormone levels of the ganders at various reproductive stages are presented. This study demonstrated that the testicular weight, the short diameter of the testis, and the testicular weight in the breeding season accounted for the percentage of the carcass weight, illustrating a curvilinear response. The egg-laying period of geese is from the beginning of October to the end of May of the following year, and its most productive period is from January to March in Taiwan [4]. This finding indicates that the growth rate of testicles of male geese is similar to the egg-laying curve of female geese. This phenomenon may have a significant impact on the fertilization rate of breeding geese.

Tome [22] suggested that the body weight of ruddy ducks before laying eggs could increase by 21% of the original body weight, which mainly accumulates fat for the energy source required for laying eggs.

Bogenfürst et al [5] implied that feeding geese should be restricted to make their body weight less than 4.75 kg, which could increase the number of eggs laid per goose and the fertilization rate of eggs in the next laying period. Free-feeding birds can result in lower egg production, poor feed efficiency, heavier body weight, and heavier egg weight [23]. Renema et al [15] indicated that ovarian weight was positively correlated with body weight and abdominal fat mass. The number of 2 to 3 cm follicles in female geese in the first month after laying is significantly higher than that in the second and first months before laying and in the fourth and fifth months after laying (1.4 vs 0, 0.40; 0.60, 0 and 0.20 egg/bird; p = 0.0074), implying a quadratic equation (p<0.001). The percentage of ovarian weight to carcass weight of female geese in the second and third months after laying was significantly higher than that in the second month before laying and the third and fourth months after laying (2.2 and 3.21 vs 0.08, 0.21, and 0.11%; p = 0.0139), implying a quadratic equation (p<0.01). The ovary weights of hens during the laying period were 200% heavier than that of unlighted hens before laying, whose fallopian tube weights were also significantly increased [17]. Guangdong geese have 10 small and large yellow follicles before laying eggs, and about 8 of them will ovulate and be laid [24]. The ovary weight of hens before laying was significantly lighter when laying the first egg, and the number of large follicles in hens when laying the first egg was significantly higher than that before laying [25]. The number of small and large yellow follicles at the beginning of egg-laying of geese was significantly higher than that at the end of hatching, and the number of small and large yellow follicles at the beginning and peak of egg-laying of geese was also significantly higher than that at the end of egg-laying [24]. The above research shows that the weight and length of the reproductive tract of poultry increase as they enter the laying stage. The data of this experiment are similar to the above data. The reproductive system of the female goose has apparent changes in preparation for laying eggs in the laying stage before the end of egg-laying. The reproductive system also has apparent shrinkage; in the number of follicles, it can be seen that the number of follicles at the peak of egg-laying is higher than that at the end of egg-laying.

The growth equation involved the body weight, weight gain, feed consumption, and feed conversion efficiency of white and grey geese from China [26]. The API range of the pink-footed goose used by Madsen and Klaassen [8] can be broadly classified as 0 to 7, which is less difficult in distinguishing the abdominal classification of the Roman. Observing the relationship between the male goose and the reproductive system using API and ASI is not easy. However, these methods can be applied to the reproductive system observation of female geese. The correlation between ASI and the reproductive system of female geese during the laying period is high, and the net correlation coefficient between it and the laying rate is 0.790, indicating that the ASI observation in female geese during the laying period can be used to predict the reproductive system of female geese during the laying period. The system changes and their correlation with egg production are the findings of this study. In the future, this method can be used to predict the commencement of female goose egg production.

## CONCLUSION

The goose observations can take advantage of ASI during egg-laying. It can predict the reproductive system development during laying and determine when breeding geese begin to lay eggs. In particular, female ASI was positively correlated with the reproductive tract length and egg production per female. Based on the above findings, the ASI classification method was more objective and easy to distinguish. This scoring method has a high correlation with the number of eggs laid by each goose and the length of the reproductive tract.

## Figures and Tables

**Table 1:** Effect of reproduction system of breeding geese at various egg production months

Item	Laying period								L	Q

	−2	−1	0	1	2	3	4	5		

	Month									

	10	11	12	1	2	3	4	5		
	---------------------------------------------------------------------------------- Male ----------------------------------------------------------------------------------									
Body weight (kg/bird)	5.25±0.27	5.16±0.27	5.85±0.27	5.47±0.27	4.61±0.24	5.19±0.27	5.37±0.27	4.80±0.27	NS	NS
Testicle weight (g/bird)	5.70±2.60	10.6±2.60	12.5±2.60	12.4±2.60	14.7±2.60	18.5±2.60	13.5±2.60	8.32±2.60	NS	^**^
Testicle width (mm/bird)	12.7±1.68^c^	18.8±1.88^ab^	19.0±1.68^a^	18.4±1.68^ab^	20.8±1.53^a^	20.4±1.53^a^	19.6±1.68^a^	14.0±1.68^bc^	NS	^***^
Testicle length (mm/bird)	19.2±12.8	27.0±14.3	27.2±12.8	24.5±12.8	57.2±11.7	28.4±12.8	26.2±12.8	19.7±12.8	NS	NS
Penis length (cm/bird)	8.72±1.53	11.6±1.53	15.2±1.53	10.4±1.53	12.4±1.53	12.2±1.53	13.6±1.53	10.8±1.53	NS	NS
Testicle weight (% of FDBW)	0.11±0.05^d^	0.22±0.05^bcd^	0.22±0.05^bcd^	0.23±0.05^abcd^	0.33±0.044^ab^	0.37±0.05^a^	0.25±0.05^abc^	0.19±0.05^cd^	NS	^**^
	--------------------------------------------------------------------------------- Female --------------------------------------------------------------------------------									
Body weight (kg/bird)	4.36±0.36^bc^	5.33±0.36^ab^	6.09±0.36^a^	5.85±0.36^a^	5.35±0.40^ab^	4.79±0.36^bc^	4.08±0.36^c^	4.07±0.36^c^	NS	NS
Length of genital tract (cm/bird)	20.1±10.2^e^	37.1±10.2^cde^	62.0±10.2^abc^	83.7±10.2^a^	78.0±11.4^ab^	69.8±10.2^ab^	30.4±10.2^de^	50.8±10.2^bcd^	NS	^***^
Genital tract weight (g/bird)	4.16±24.4^d^	27.6±24.4^cd^	90.4±24.4^bc^	107±24.4^ab^	181±27.3^a^	9.76±24.4^d^	23.1±24.4^cd^	62.2±24.4^bcd^	NS	^***^
Ovary weight (g/bird)	3.24±31.3^d^	61.7±31.3^bc^	99.4±31.3^abc^	128±31.3^ab^	158±35.0^a^	9.38±31.3^cd^	4.50±31.3^d^	29.5±31.3^cd^	NS	^**^
Follicle number 2 to 3 cm, egg	0±0.25^c^	0.40±0.25^bc^	0.60±0.25^bc^	1.40±0.25^a^	0.75±0.28^abc^	0.80±0.25^ab^	0±0.25^c^	0.20±0.25^bc^	NS	^***^
Follicle number 3 to 4 cm, egg	0±0.45	0.400±0.45	1.40±0.45	1.00±0.45	1.00±0.51	1.00±0.45	0±0.45	0.400±0.45	NS	^*^
Follicle number 4 to 5 cm, egg	0±0.54	1.20±0.54	1.00±0.54	1.20±0.54	0.75±0.60	0.60±0.54	0±0.54	0.20±0.54	NS	NS
Follicle number 5 to 6 cm, egg	0±0.17	0±0.17	0±0.17	0.600±0.17	0±0.19	0.40±0.17	0±0.17	0±0.17	NS	NS
Genital tract weight (% of FDBW)	0.11±0.57^bc^	0.57±0.57^bc^	1.57±0.57^bc^	1.91±0.57^b^	3.68±0.64^a^	0.22±0.57^c^	0.58±0.57^bc^	1.67±0.57^bc^	NS	^*^
Ovary weight (% of FDBW)	0.08±0.61^c^	1.30±0.61^bc^	1.70±0.61^abc^	2.20±0.61^ab^	3.21±0.68^a^	0.21±0.61^c^	0.11±0.61^c^	0.80±0.61^bc^	NS	^**^

L, linear; Q, quadratics; SEM, standard error of means for treatment; NS, no significant; FDBW, eighteen-h feed-deprived body weight.

a–e Means without the same superscripts within the same row under treatment differ significantly (p<0.05). The least-square means (LSMEANS) method was used to determine the difference between the means of each treatment.

* p<0.05.

** p<0.01.

*** p<0.001.

**Table 2:** Effect of laying period on abdomina profile score and abdominal sagging index of breeding goose

Item	Laying period							

	−2	−1	0	1	2	3	4	5
Abdominal profile index	5.36±0.075^c^	5.64±0.077^b^	5.66±0.079^b^	5.89±0.082^a^	5.90±0.085^a^	5.78±0.088^ab^	5.73±0.092^ab^	5.85±0.096^ab^
abdominal sagging index	3.34±0.086^c^	3.39±0.088^c^	3.54±0.091^bc^	3.89±0.094^a^	3.86±0.098^a^	3.94±0.101^a^	3.68±0.106^ab^	3.68±0.110^ab^

SEM, standard error of means for treatment.

a–c Means without the same superscripts within the same row under treatment differ significantly (p<0.05).

**Table 3:** Partial correlation coefficients among abdominal profile score and abdominal sagging index and reproduction system in gander

Item	ASI	TEW	TESTL	TESTW	PSL
API	0.93^***^	0.44	0.22	0.49	0.36
ASI		0.04	0.45	0.11	−0.30
TEW			0.50	0.90^**^	0.57
TESTL				0.63^†^	0.31
TESTW					0.68^†^

API, abdominal profile index; ASI, abdominal sagging index; TEW, testicle weight (g/bird); TESTL, testicle length (mm/bird); TESTW, testicle width (mm/bird); PSL, penis length (cm/bird).

† p<0.1;

* p<0.05.

** p<0.01.

*** p<0.001.

**Table 4:** Partial correlation coefficients among abdominal profile score and abdominal sagging index and reproduction system in goose

Item	API	ASI	LG	GTW	OW	F2C	F3C	F4C	LN
BW	0.402	0.363	0.805^*^	0.705^†^	0.877^**^	0.878^**^	0.848^**^	0.837^**^	0.017
API		0.840^**^	0.786^*^	0.633^†^	0.528	0.597	0.482	0.335	0.488
ASI			0.815^*^	0.452	0.341	0.664^†^	0.509	0.162	0.790^*^
LG				0.716^*^	0.732^*^	0.905^**^	0.856^**^	0.613	0.466
GTW					0.930^***^	0.542	0.596	0.436	0.160
OW						0.696^*^	0.681^†^	0.711^*^	0.039
F2C							0.772^*^	0.763^*^	0.309
F3C								0.703^†^	0.269
F4C									−0.074

API, abdominal profile index; ASI, abdominal sagging index; LG, length of genital tract (cm/bird); GTW, genital tract weight (g/bird); OW, ovary weight (g/bird); F2C, follicle number 2 to 3 cm (egg/bird); F3C, follicle number 3 to 4 cm (egg/bird); F4C, follicle number 4 to 5 cm (egg/bird); LN, egg number per goose.

† p<0.1;

* p<0.05.

** p<0.01.

*** p<0.001.
